
JOURNAL INFORMATION
==============================
NLM Title Abbreviation: Inter Econ
Journal ID: Inter Econ
NLM Journal ID: 101086399

Inter Economics

ISSN: 0020-5346

ARTICLE INFORMATION
==============================
PMCID: PMC8176658
PMID: 34103764
DOI: 10.1007/s10272-021-0976-7
Article ID: 976
Article version: 1
Subjects: Letter from America

US Support for a WTO Waiver of COVID-19 Intellectual Property

Contreras Jorge L. jorge.contreras@law.utah.edu

grid.223827.e 0000 0001 2193 0096 S.J. Quinney College of Law, University of Utah, 383 South University St E, Salt Lake City, UT 84112 USA
Electronic publication date: 2021 Jun 4
Print publication date: 2021
Volume: 56
Issue: 3
First page: 179
Last page: 180
Copyright: © The Author(s) 2021
License: Open Access: This article is distributed under the terms of the Creative Commons Attribution 4.0 International License (https://creativecommons.org/licenses/by/4.0/).
Open Access funding provided by ZBW — Leibniz Information Centre for Economics.
License URL: https://creativecommons.org/licenses/by/4.0/

issue-copyright-statement: © The Author(s) 2021

==============================

On May 5, 2021, United States Trade Representative (USTR) Katherine Tai announced that the U.S. would support the "waiver of IP [intellectual property] protections on COVID-19 vaccines" that was introduced at the World Trade Organization (WTO) in October 2020 by the governments of India and South Africa. The announcement came as a surprise to many, as it represents a reversal of the strong stance that the U.S. has taken on patents and other intellectual property issues since at least the Reagan Administration. As such, the move was cheered by proponents of broader access to medicines around the world, while the biopharmaceutical industry, along with the governments of Germany and France, warned of dire economic consequences that could follow the elimination of patents on critical medical technologies. But what, precisely, does U.S. support for this global IP waiver mean, and what are its likely effects?

The WTO is the treaty organization under which the principal international agreement concerning intellectual property was adopted in 1995 – the Trade-Related Aspects of Intellectual Property Rights (TRIPS) Agreement. The TRIPS Agreement requires WTO member states to enact laws protecting various forms of intellectual property including patents, copyrights and trade secrets, and to impose minimum levels of protection for these rights (e.g. a 20-year term for patents). If a country is believed to have violated the TRIPS Agreement, another WTO member state, or an aggrieved private company, may bring a claim to the WTO. Though the WTO itself has no authority to impose penalties for violation of its agreements, a finding of liability by a WTO tribunal is usually viewed as justifying the imposition of trade sanctions by the complaining party's country.

Though the TRIPS Agreement requires WTO member states to issue patents for eligible inventions and to give national treatment to all patent applicants, it also permits states to authorize third parties to operate under issued patents to ensure the domestic supply of a patented product, provided that the patent owner is paid adequate remuneration. This practice is called "compulsory licensing". In 2001, a group of member states adopted the supplemental "Doha Declaration" that permits states to grant compulsory licenses for the export of pharmaceutical products to meet public health needs in other countries. Over the past 30 years, countries including India, South Africa, Brazil and Thailand have issued compulsory patent licenses under these TRIPS flexibilities to increase local supplies of drugs for HIV/AIDS, cancer and heart disease. In most cases, the U.S. and other Western states have opposed these measures.

Beginning in March 2020, countries including Chile, Ecuador, Canada, Germany, France and Israel enacted, or seriously considered, compulsory licensing and access measures to address the emerging COVID-19 pandemic. Indonesia and Brazil have more recently taken such measures. Then, in October, the governments of South Africa and India requested that the WTO issues a waiver providing that countries not be deemed to violate TRIPS for suspending, in their countries, the enforcement of patents, copyrights, industrial designs, and trade secrets "in relation to prevention, containment or treatment of COVID-19." In effect, if enacted, the waiver would prevent other WTO member states from bringing trade-related challenges against these countries at the WTO.

The proposed WTO waiver is notable because it would expand existing TRIPS compulsory licensing flexibilities beyond patents by authorizing compulsory licensing of copyrights, industrial designs and trade secrets for any use relating to COVID-19. This expansion is viewed as critical because vaccines are complex and volatile products, so patents alone are generally not sufficient to enable a manufacturer to reproduce another company's product. Trade secrets and manufacturing know-how, as well as raw materials and suitable production facilities, are essential. The proposed WTO IP waiver would allow a country that wished to suspend trade secret protection for COVID-19 technology to do so without violating the TRIPS Agreement and incurring international trade sanctions. Such a country could also, presumably, mandate that foreign companies disclose their proprietary manufacturing and testing information to local producers under a compulsory license. The details of this disclosure requirement, and any compensation payable to the originator of the information, would need to be worked out in whatever waiver is eventually adopted by the WTO, but the prospect of a mandatory trade secret transfer – something that would be unprecedented in the international arena – is potentially significant. At the moment, Brazil is the only major country that has proposed such a scheme, though the passage of a WTO waiver could encourage other countries to do so.

The USTR's statement indicates that the U.S. will negotiate at the WTO for a broadly acceptable waiver of COVID-related IP rights and, if such a waiver is approved at the WTO, the U.S. will not pursue trade sanctions against countries issuing COVID-related compulsory licenses. However, this commitment would have little effect on U.S. vaccine producers that do not, themselves, have material operations overseas. A state's authority over a private company is only effective to the extent that the company possesses assets within the state. Only the U.S. government could require a U.S.-based company to disclose its trade secrets, and the prospect of this happening is slim. It is one thing for the U.S. to agree not to seek sanctions against other countries that impose COVID-19 compulsory licensing regimes, but a very different thing for the U.S. to issue a compulsory licensing order of its own, particularly in the area of trade secrets, where it would be met with significant internal opposition. In the end, it is likely that, even if the WTO does adopt an IP waiver, only a handful of countries with domestic generics industries – Brazil, India, Thailand, South Africa, Canada – are likely to avail themselves of the opportunity to impose compulsory licensing regimes that include both patents and trade secrets.

In the end, the impact of a WTO IP waiver on international vaccine supplies will depend in large part on how other countries elect to implement compulsory licensing rules under the waiver, and whether they can effectively require the transfer of confidential manufacturing, testing and safety information to local producers. Ultimately, the threat of such governmental action could encourage companies to engage in voluntary knowledge transfer to alleviate global supply shortages, which might be the greatest benefit of the WTO IP waiver. After all, even if a company is legally required to "share" its proprietary trade secrets and manufacturing know-how with others, there are countless ways to delay and subvert the effective transfer of knowledge. In practice, the most effective technology sharing programs are supported by voluntary action rather than governmental compulsion.

Nevertheless, U.S. support for an international IP waiver is an important gesture toward global cooperation in a time of crisis. It represents a significant reversal of prior U.S. policy under both Republican and Democratic administrations, which roundly condemned the compulsory licensing of IP under most circumstances. As such, the USTR's statement should be applauded.

Jorge L. Contreras, University of Utah, Salt Lake City, USA.

